# Efficacy and Safety of Percutaneous Transhepatic Lithotripsy Using SpyGlassDSTM Cholangioscopy for the Treatment of Difficult Stones

**DOI:** 10.3390/diagnostics15091060

**Published:** 2025-04-22

**Authors:** Salvatore Alessio Angileri, Giuseppe Pellegrino, Carolina Lanza, Jacopo Pozzi, Marco Costa, Matilde Pavan, Pierpaolo Biondetti, Serena Carriero, Velio Ascenti, Gaetano Valerio Davide Amato, Pierluca Torcia, Anna Maria Ierardi, Gianpaolo Carrafiello

**Affiliations:** 1 Department of Diagnostic and Interventional Radiology, Foundation IRCCS Cà Granda—Ospedale Maggiore Policlinico, Via Francesco Sforza 35, 20122 Milan, Italy; alessioangileri@gmail.com (S.A.A.); pierpaolo.biondetti@policlinico.mi.it (P.B.); serena.carriero@unimi.it (S.C.); velio.ascenti@unimi.it (V.A.); pierluca.torcia@policlinico.mi.it (P.T.); annamaria.ierardi@policlinico.mi.it (A.M.I.); 2 Department of Oncology and Hemayo-Oncology, Università degli Studi di Milano, 20122 Milan, Italy; giuseppe.pellegrino@unimi.it (G.P.); gaetano.amato@unimi.it (G.V.D.A.); 3 Postgraduate School in Radiodiagnostics, Università degli Studi di Milano, 20122 Milan, Italy; jacopo.pozzi@unimi.it (J.P.); marco.costa@unimi.it (M.C.); matilde.pavan@unimi.it (M.P.)

**Keywords:** percutaneous transhepatic cholangioscopy (PTC), hepatobiliary disease, biliary stones, balloon bilioplasty, cholangitis, percutaneous transhepatic biliary drainage (PTBD), SpyGlassDSTM

## Abstract

**Background/Objectives**: the aim of this study was to evaluate the safety and efficacy of percutaneous transhepatic lithotripsy using the SpyGlassDSTM cholangioscopy system for the treatment of difficult stones. **Methods**: Retrospectively, all patients treated with percutaneous transhepatic lithotripsy using SpyGlassDSTM cholangioscopy system were analyzed. As primary outcome measures, the following data were assessed: the presence of a previous history of the hepatobiliary disease, location of stones, reasons for the choice of the procedure, previous balloon bilioplasty, type of pre-procedural imaging, procedural time, technical success, clinical success, and post-procedural complications (according to CIRSE classification). Clinical success was considered “primary” when achieved with a single treatment, and “secondary” if more than one treatment was required in the duration of follow-up. **Results**: 10 patients (6 males and 4 females, mean age = 64 years, SD = 22), all with cholangitis due to gallstones, underwent 11 PTL procedures using SpyGlassDSTM. Technical and clinical successes were achieved in all patients (100%). Primary success was observed in 4/10 (40%) patients, while the remaining 6/10 (60%) patients undergoing re-treatment, and all showed secondary success (100%). No periprocedural complications were observed. In 10/11 procedures (90%), no relevant adverse events were recorded within the first thirty days of follow-up. In 1/11 case (9%), mild complications (grade I according to CIRSE classification) were registered in the following days after the procedure (<30 days). **Conclusions**: in conclusion, the treatment of percutaneous transhepatic lithotripsy using SpyGlassDSTM cholangioscopy of difficult stones has been demonstrated as efficient and safe treatment.

## 1. Introduction

The prevalence of gall bladder lithiasis in Europe is reported between 5.9 and 21.9% [1]. Up to 20% of individuals with gallbladder stones also have synchronous common bile duct stones (CBDSs). CBDSs can result in potentially fatal side effects, including acute pancreatitis or acute cholangitis, thus are important to detect and treat [2].

CBDS therapy involves common bile duct (CBD) clearance, and with consideration that most cases of CBDSs are caused by gallstone migration from the gallbladder; hence, cholecystectomy became a further necessary subsequent intervention [2].

Endoscopic Retrograde Cholangiopancreatography (ERCP) with either sphincterotomy or lithotripsy with balloon/Dormia basket-assisted stone extraction is the primary procedure for treating biliary lithiasis [3]. This technique has been widely used for the diagnosis and management of a spectrum of benign and malignant pancreaticobiliary disorders, particularly in cases of common bile duct (CBD) stones and biliary obstruction from either benign or malignant etiologies. ERC followed by endoscopic sphincterotomy is the most often utilized approach for visualizing and treating CBD stones [4,5,6]. The technique involves endoscopically identifying the papilla major (Vater papilla), cannulating it for ERC and sphincterotomy, and extracting CBDSs using a Dormia Basket or balloon. Lithotriptors are made up of a big hard-wire basket and an extra metal spiral sheath that is inserted over the basket into the CBD. When in position, the metal basket is opened to trap the stones before being brought back to the lithotriptor’s exterior hardduct, where lithotripsy takes place [2].

Stone impaction in the CBD and CBDS sizes greater than 3 cm [7], which prevents stones from being caught, are factors linked to lithotripsy failure.

Electrohydraulic and laser systems represent different lithotripsy methods used for the management of biliary stones. Their first applications date back to late 1990s, and involved both the treatment of CBD and intrahepatic/gallbladder stones [8,9]; their stone fragmentation mechanisms are, respectively, based on high-energy shock waves and laser lithotripters, such as the holmium laser (holmium:yttrium aluminum garnet or Ho:YAG), which is the most recent, though its implementation is still restricted, since it requires expensive equipment [2].

One of the most difficult aspects of treating biliary stones is represented by patients whose anatomy has changed after surgery. In those situations, anatomical alterations, such as those that occur after gastric bypass or biliary-enteric diversion, may make standard endoscopic retrograde cholangiopancreatography (ERCP) impractical [10].

In addition, the rendezvous technique, which includes both antegrade and retrograde approaches, has emerged as a significant strategy for handling patients with changed anatomy. This procedure makes it easier to access difficult-to-reach biliary stones by first conducting percutaneous access to the bile duct, and then connecting it to the duodenum or papillary duct, allowing for lithotripsy or stone removal using a combined approach [11]. In this context, Percutaneous Transhepatic Lithotripsy (PTL), when combined with SpyGlass DS™ cholangioscopy, emerges as an efficient method to treat complex stones, minimizing the necessity for other invasive interventions.

The evolution of direct peroral visualization techniques has significantly advanced the field of cholangioscopy. While direct visualization of the bile duct has been available since the 1970s, technological advancements in recent years have greatly enhanced its diagnostic and therapeutic benefits [12]. Currently, three main cholangioscopy systems are available: single-operator, dual-operator, and direct cholangioscopy, with each offering specific advantages in terms of usability, image quality, and diagnostic precision [13].

In particular, single-operator cholangioscopy (SOC) represents a breakthrough in biliary stone management and the evaluation of biliary strictures. Unlike earlier fiberoptic cholangioscopes, which required two trained endoscopists and were cumbersome with suboptimal image resolution, SOC, particularly with the introduction of the SpyGlass™ system (Boston Scientific, Natick, MA, USA), has facilitated more efficient, high-resolution imaging and intervention. The first-generation SpyGlass, introduced in 2007, was a reusable single-operator fiberoptic scope that included a dedicated channel for accessory instruments and irrigation capabilities, significantly improving maneuverability and ease of use compared to its predecessors [14].

In 2015, the second-generation SpyGlass DS was introduced, incorporating a digital imaging system with four times the resolution of the original version, and a wider field-of-view (110° vs. 70°), leading to enhanced visualization of biliary structures. Additionally, it featured a redesigned accessory channel, which further simplified the deployment of biopsy forceps and electrohydraulic or laser lithotripsy probes, making the procedure more efficient and accessible [12].

Electrohydraulic lithotripsy (EHL), originally developed for the treatment of kidney stones, has been successfully adapted for biliary applications. Utilizing a charge generator and a bipolar probe, EHL produces a spark that generates vapor plasma, resulting in high-energy shock waves that fragment the stones. The integration of this technology with the SpyGlass system has enhanced stone fragmentation, particularly in cases where conventional ERCP techniques are inadequate [15].

Clinical studies have demonstrated the efficacy of SpyGlass-guided interventions. A multicenter registry study involving 297 patients across the United States and Europe reported a procedure success rate of 89%, with successful stone fragmentation and removal initiation in 92% of cases [14]. Furthermore, SpyGlass-directed biopsy has proven to be superior to traditional brush cytology, with studies indicating a significantly higher diagnostic accuracy for biliary strictures when compared to blind biopsies [16,17,18].

The benefits and drawbacks of peroral endoscopy are common to other endoscopic treatments. Even though it is generally accepted that average endoscopists may easily reach the second duodenum by endoscopy in average individuals, some circumstances can make this maneuver challenging.

For example, some anatomical constraints (e.g., strictures downstream of the stone, duodenal diverticulum at the papilla of Vater, prior surgical interventions such as Billroth II or Roux-en-Y reconstruction), or the presence of impacted stones, make ERCP a technique with suboptimal results, or even impracticable [19].

Alternative approaches include open or laparoscopic surgery, which are associated with a higher rate of complications [20]. Other less invasive options include extracorporeal shock wave lithotripsy (ESWL) or percutaneous transhepatic mechanical approaches utilizing holmium laser or electrohydraulic systems (EHL) [20]. According to the most recent guidelines from the European Society of Gastrointestinal Endoscopy, these alternatives are valuable options for patients in whom ERCP has failed or is not feasible [21].

Percutaneous radiographic stone extraction is currently recommended for the small number of individuals in whom endoscopic methods fail or are impossible to perform. To gain biliary access, a percutaneous transhepatic cholangiography (PTC) drain is inserted. Catheter procedures are then conducted under fluoroscopic guidance [22].

Another chapter of gallstone disease is represented by hepatolithiasis, defined as the presence of stones within the intrahepatic bile ducts proximal to the right and left hepatic ducts. The incidence of hepatolithiasis is high in East Asian countries and rare in Western countries.

Parasitic infestation has often been thought to be a major cause of hepatolithiasis and infestation, as parasites have been detected in up to 30% of patients with hepatolithiasis [23,24].

Hepatolithiasis is more common in the left lobe because the left hepatic duct coalesces with the CBD at an acute angle, which tends to induce bile stasis when associated with a biliary stricture. The main complications are represented by biliary strictures, liver abscess, liver cirrhosis, and cholangiocarcinoma [24].

Complete stone removal and preventing recurrent cholangitis are the main objectives of treatment for hepatolithiasis. Currently available treatment options include both surgical procedures, like hepatic resection, and nonsurgical procedures, like percutaneous transhepatic cholangioscopic lithotripsy (PTCSL). PTCSL has been frequently used with a fair success rate [25,26].

Although recurrent cholangitis is rather prevalent in PTCSL, the rate of total stone removal was observed to be comparably high in both therapies [27,28].

Hepatic resection has been considered the definite treatment for hepatolithiasis, but is associated with an post-hepatectomy infection rate of 23.8%, which is higher in hepatolithiasis than in other hepatic malignancies [22,29].

Given the limited number of studies on this advanced cholangioscopy technique, the present work aims to showcase the efficacy and safety of percutaneous transhepatic cholangioscopy with the SpyGlass DS system in patients with “difficult” bile stones. This includes those with anatomical variations or strictures where standard ERCP is not viable. The study will also delineate the procedural technical steps required for successful execution, contributing to the growing body of evidence supporting the expanded use of SpyGlass-guided cholangioscopy in pancreaticobiliary disease management.

## 2. Material and Methods

### 2.1. Patients

From 1 January 2022, to 31 December 2023, all patients affected by biliary cholangitis caused by intra- or extrahepatic lithiasis, treated with percutaneous transhepatic lithotripsy (PTL) using SpyGlassDSTM cholangioscopy system, were retrospectively reviewed (Table 1).

All subjects consented to be included in the study, which was conducted per the Declaration of Helsinki. The treatment modality was chosen after a multidisciplinary meeting involving interventional radiologists, endoscopists, and liver surgeons. The therapeutic option was electively selected in the presence of the following conditions: previous surgeries (hepaticojejunostomy, Billroth II anastomosis, gastrectomy, biliopancreatic diversion, gastric bypass), common bile duct stenosis, anatomical variants of the bile duct, or previous unsuccessful endoscopic retrograde cholangiopancreatography (ERCP). All procedures were performed by interventional radiologists in a dedicated angiography room.

### 2.2. Outcome Measures

The following data were collected: the presence of a previous history of the hepatobiliary disease, location of stones, reasons for the choice of the procedure, previous balloon bilioplasty, type of pre-procedural imaging, procedural time, technical success, and clinical success after one or more procedures (comorbidities, prior surgery, or endoscopic attempt), post-procedural complications (according to CIRSE classification) [30] and duration of follow-up.

Technical success was defined as the removal of target stones and the restoration of biliary tract flow. Clinical success was defined as the resolution of symptoms at a 30-day follow-up, considered “primary” when achieved with a single treatment, and as “secondary” if more than one treatment was required. Follow-up was performed with a hospital visit by the referring physician one month after the procedure. MRI-cholangiography was repeated in the presence of symptoms suggestive of recurrent cholangitis.

### 2.3. Patient Selection Criteria: Clinical and Imaging Indications

The indication to treatment was based on clinical signs and symptoms of cholangitis and the presence of biliary stones, assessed by a Magnetic Resonance Cholangiopancreatography (MRCP), concerning the stone size, the location (intra-hepatic or extrahepatic) and the quantity (single or multiple).

### 2.4. Statistical Analysis

Given the small sample size and the study’s observational nature, only descriptive analyses were obtained for all variables assessed in the study population.

Mean and standard deviation were used for normally distributed variables, mean and interquartile range for skewed distributions, and proportions for categorical variables. Whenever relevant, 95% confidence intervals (95%CI) were calculated. No group comparisons were planned.

### 2.5. Procedure

All patients were submitted to percutaneous transhepatic biliary drainage (PTBD) placement (8 F pigtail catheter) 5–7 days before PTL due to the presence of cholangitis. The rationale for positioning the PTBD was to decompress the biliary tree and relieve biliary pressure.

Fluoroscopic and ultrasound guidance were used to access, respectively, the right and left sides. The internal-external PTBD was preferentially chosen, but in the case of serrate stenosis of the common bile duct due to the presence of stones, an external PTBD was used.

The PTL was undertaken only after an improvement of cholangitis, as demonstrated by clinical and laboratory assessments.

The biliary tract entry point was selected based on the location of the stone, using the rule of contralateral access (which means choosing the right side when the stone is to the left and vice versa).

All procedures were performed in an angiography room under fluoroscopic guidance (Philips Azurion 7 B20/15, Philips, Best, The Netherlands) by a 10-year-experienced interventional radiologist. An antibiotic prophylaxis consisting of 2 g of cefazolin sodium (Ancef, SmithKline Beecham Pharmaceuticals, Philadelphia, PA, USA) was administered before each procedure.

Both PBD and PLT procedures were performed with anesthesiologist support and moderate sedation with midazolam 0.07–0.08 mg/kg (Ipnovel15, Roche, Milan, Italy), propofol 0.5–2.0 mg/kg/h (Diprivan, AstraZeneca S.p.A., Caponago, Italy) and fentanyl 1–2 mg/kg (Fentanest; Pharmacia & Upjohn, Milan, Italy). Electrocardiographic trace, heart rate, blood pressure, respiratory frequency, and oxygen saturation were continuously monitored during the procedure.

After the prior placement of 8F pigtail biliary drainage, a 10 F shift (Terumo, Tokyo, Japan) was used to insert a 10 F flexible colonoscopy SpyGlass DSTM (Boston Scientific Marlborough, MA, USA). A preliminary cholangiography with a 5 F vertebral catheter (Cordis) was used to assess the biliary tree after the decompression obtained from the previous PTBD placement.

The SpyGlass Direct Visualization system includes a light source, irrigation tube, access and delivery catheter (SpyScope), and optical probe (SpyGlass).

The optical probe is composed of a one-use SpyScope 10 Fr access with a delivery catheter equipped with a 1.2 mm diameter working channel (made of dedicated irrigation channels) and a reusable SpyGlass Fiber Optic Probe that provides 6000-pixel images and a 70-degree field of view. The device was introduced over a 0.035” guidewire. The catheter tip can be deflected in four routes with an inclination angle of at least 30°. The second structure is an electrohydraulic system, introduced from the dedicated canal of the choledoscopy for fragmenting the stones.

The generator (AUTOLITH, Northgate Technologies Inc., Elgin, IL, USA) is connected to a probe (Northgate Technologies Inc., Elgin, IL, USA) with a caliber of 1.9 F.

The generator settings can be adapted depending on the type of stone (a minimum of 10 pulses per second and a power output of 60 W).

**Figure 1:**
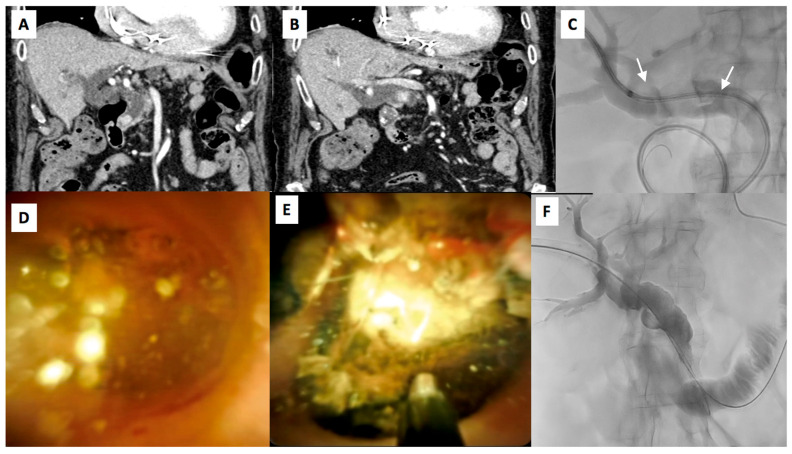
A 65-year-old female affected by extra-hepatic principal biliary duct lithiasis, as showed on CECT images on and coronal plane (**A**,**B**) demonstrating the presence of two hyperdense bile stones of 15 mm and 12 mm in the middle and in distal tract of common bile duct. (**C**) Pre-procedural cholagiography confirms the presence of biliary stones (white arrows). (**D**,**E**) Choledoscopic image of biliary stone and probe positioning before and during lithotripsy. (**F**) Final cholangiography demonstrates the removal of biliary stones.

**Figure 2:**
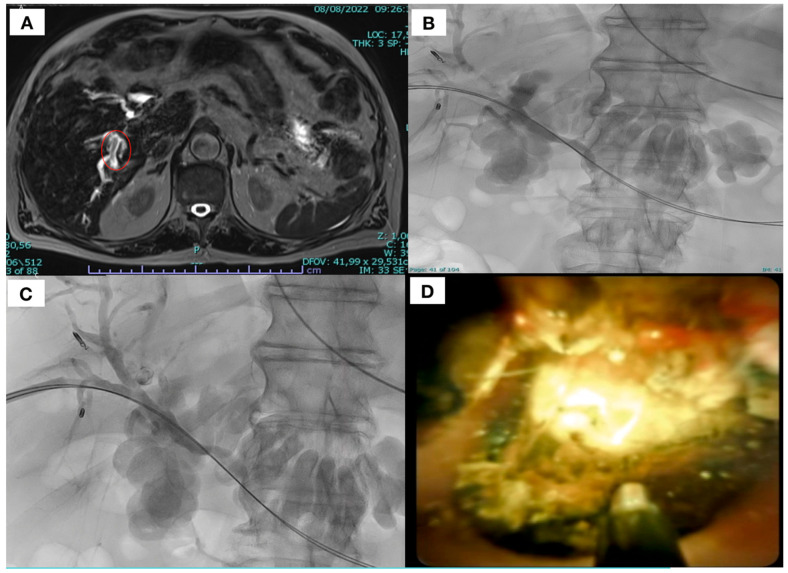
A 45-year-old affected by intra-hepatic lithiasic filling defects inside as demonstrated in heavily T2 weight haste sequence (**A**) (red circle). (**B**) Pre-procedural cholangiography showed the presence of multiple biliary stones as filling defects with prevalence on the right side and post-procedural (**C**) cholangiography, confirming the removal of right duct intrahepatic lithiasis; (**D**) choledoscopic image of biliary stone and probe positioning before lithotripsy.

When a charge is applied over the electrodes at the probe’s tip, a spark causes the surrounding fluid to swell, resulting in an oscillating pressure shock wave sufficient to fragment most of the stones. Immediately after every litotrypsy fragmentation, a balloon-based bilioplasty was performed to both remove residual biliary debris and to dilate eventual strictures. Balloon size is chosen on the basis of different factors that range from the location of the stones (intra or extrahepatic) and the caliber of the biliary structure of interest; according to the most recent European Society of Gastrointestinal Endoscopy (ESGE) guidelines [31], a balloon size of 6–8 mm is a typically safe and effective range for most patients. The bilioplasty consists of a gradual dilation of the biliary chosen structure, starting with smaller balloons (4–6 mm) up to the final balloon, sized up to 10 mm in case of CBD stones, for a maximum time of 3 min to achieve the optimal combination of therapeutic effect and procedural safety. At the end of the procedure, a 10–12 F internal-external biliary drainage tube was left. Before the biliary drainage was removed (two to three days after PTL), another cholangiography was performed in each patient to demonstrate the patency of the biliary tree (Figure 1 and Figure 2).

## 3. Results

### 3.1. Patients

The population included 10 patients (6 males and 4 females, mean age = 64 years, SD = 22), all with cholangitis due to gallstones, who underwent 11 PTL procedures using SpyGlassDSTM.

In our study cohort of 10 patients, 2/10 (20%) had a previous history of hepatobiliary disease: one with chronic liver disease progressing to cirrhosis and another with secondary biliary cirrhosis following multiple orthotopic liver transplantations. In total, 3/10 (30%) patients had intrahepatic lithiasis, while 7/10 (70%) had extrahepatic lithiasis.

In 6/10 (60%) patients, ERCP was previously attempted with no success or was contraindicated by the gastroenterologist in the first instance because of either complex duodenal anatomy or previous surgery that caused a changed anatomy.

The remaining 4/10 (40%) presented with intrahepatic stones with normal anatomy and no history of surgical procedures, but gained the consensus of the multidisciplinary team to undergo the percutaneous procedure, which was deemed more appropriate considering the stones’ location and pre-procedural assessment anatomical data.

In 1/10 (10%) patients, contrast-enhanced CT was used as pre-procedural imaging, while in the other 9/10 (90%) patients, MRI with colangio-MRI was performed.

### 3.2. Procedure Details

Technical success and clinical success were achieved in all patients (100%). Primary success was observed in 4/10 (40%) patients, while the remaining 6/10 (60%) patients undergoing re-treatment and all showed secondary success (100%). Five of the six patients requiring retreatment underwent two procedures, while one received three treatment sessions.

Notably, three patients required subsequent percutaneous transhepatic biliary stenting (PTBS) within seven days after the initial procedure, one patient underwent ERCP with sphincterotomy and endoscopic balloon dilatation (EBD), and another required supplementary cholecystectomy. The patient who underwent three treatments received two PTL procedures with SpyGlassDSTM within seven days, followed by PTBS as a completion procedure.

Previous balloon bilioplasty was performed in six out of eleven (54%) procedures. The median procedural time was 107 min (40–195 min, SD: 40) (Table 1).

### 3.3. Safety and Follow-Up

No periprocedural complications were observed. In 10/11 procedures (90%), no relevant adverse events were recorded within the first thirty days of follow-up. In 1/11 case (9%), mild complications (grade I according to CIRSE classification) were registered in the following days after the procedure (<30 days). Specifically, patient #3 exhibited increased inflammatory and liver damage markers the day after the procedure, along with evidence of right pleural effusion on chest X-ray. Symptomatic treatment was sufficient during the residual convalescence period. After eight days, PTCS with stenting was performed, and the patient was discharged in stable conditions after 14 days.

In all cases, MRI-cholangiography was repeated 30 days after the last procedure, as recommended by literature works about imaging follow-up of biliary percutaneous procedures [32].

## 4. Discussion

The possibility of directly visualizing the biliary tree granted by the choice of cholangioscopy devices offers the operator invaluable benefits during the bile stone removal procedure [33]. The advanced technologies of these systems provide broad fields of view, excellent maneuverability, and utmost control over the navigation of biliary systems. Moreover, these devices can be used with both endoscopic and percutaneous access [34], the latter potentially being greatly advantageous in cases where strictures cause difficult access from the digestive system, such as in surgically altered anatomy or other anatomical characteristics (e.g., intra-diverticular or unreachable papilla).

PTL with SpyGlass cholangioscopy has drawbacks in terms of accessibility, complication risk, and invasiveness, even though it is an effective treatment for challenging stones, especially in individuals with changed anatomy. When possible, ERCP is the recommended option because it is typically less invasive and simpler to execute. Nonetheless, PTL continues to be a useful substitute in difficult situations where ERCP is not feasible [35].

A randomized trial by Li et al. confirmed the clinical efficacy of SpyGlassTM-guided lithotripsy for treating large common bile ducts and its non-inferiority compared to laparoscopic exploration, with fewer adverse events and hospital stays [36]. For this reason, these procedures can provide a safer and less demanding option for patients for whom the endoscopic approach is unfeasible or failed. An even more significant advantage is provided by the combination of the information granted by the cholangioscopic device and the imaging guide of our choice, which usually consists of fluoroscopic guidance [37].

Recent studies have demonstrated the benefit of using single-operator cholangioscopy (SOC) in patients with both difficult and missed bile duct stones [38]. Stones can be classified as difficult when they are larger than 15 mm, impacted, or located near strictures. Various locations in the bile duct network have also been described where stones may be hidden or missed using magnetic resonance cholangiopancreatography (MRCP) and ERCP [39]. Direct visualization through SOC enables more precise diagnosis and targeted wire placement, increasing the success rate of stone retrieval. Patients with recurrent cholangitis, significant dilation of the bile ducts, and unusual biliary stone presentations may particularly benefit from this approach.

The majority of patients included in our series (60%) either already underwent ERCP or an endoscopic approach was deemed impossible by the gastroenterologist; the remaining 40% presented with intrahepatic stones with normal anatomy and no history of surgical procedures, highlighting that percutaneous cholangioscopy can also be helpful in this scenario [40]. Percutaneous access to the biliary system allows both diagnostic (e.g., assessment of indeterminate stenoses) and therapeutic options thanks to the consistent versatility of the system, which can be employed in combination with stenting, drainage, or other lithotripsy procedures (laser or electrohydraulic) [41].

Additionally, advancements in SOC technology, particularly with the digital SpyGlass system, have simplified the setup process, improved imaging resolution, and enhanced the ease of using biopsy forceps. The digital system facilitates a more efficient and effective approach to stone management, reducing the need for additional ERCP procedures. This is particularly relevant in cases of complex biliary anatomy, where conventional ERCP techniques have failed [42].

A recent systematic review analyzed the performance of both cholangioscopy-assisted lithotripsy techniques and confirmed the feasibility, efficacy, and safety of both systems, with better results in larger (>3 cm) stones from laser lithotripsy [43]. On the other hand, EHL seems to have less traumatic effects on the wall of the biliary tract, with lower reported hyperemia or bleeding, making it a more suitable and safe choice in older or less fit patients [44].

Moreover, SpyGlass has also been successfully used for pancreatic duct interventions, particularly in cases of symptomatic pancreatic duct calculi. A multicenter study involving 28 patients reported a treatment success rate of 79%, demonstrating its utility in managing pancreatic duct stones when the duct is sufficiently dilated for probe insertion [45]. In another study, the use of SpyGlass pancreatoscopy in patients with suspected intraductal papillary mucinous neoplasms (IPMN) showed that 76% of main duct IPMN cases and 78% of branch duct IPMN cases were correctly identified, influencing clinical decision-making in 76% of cases [46].

The overall adverse event and mortality rates related to cholangioscopic approaches have been reported to be 6% to 17% and 0% to 0.6%, respectively [15]. The most commonly reported adverse events encountered in these procedures are bleeding or hemorrhage, followed by perforation, infection, fever, sepsis, and pancreatitis [47]. However, in our study, no periprocedural adverse events occurred. The technical success rate was 100%; however the procedures were relatively lengthy (average procedural time of 107 min), still in line with literature reports [48], a factor that may potentially be overcome with the center’s expertise in acquiring greater proficiency with the cholangioscopic system.

Percutaneous trans-hepatic lithotripsy is a more prevalent treatment option in country in East Asia, where primary choledocholithiasis and hepatolithiasis are more prevalent. In the literature, very few reports mostly case reports and case series reported, predominantly describing hepatolithiasis management [23,49,50,51,52]. In our study, we have collected both cases of CBDSs and hepatolithiasis with a cohort of 10 patients followed for 1 month after the procedure.

The current study had several limitations: it was a retrospective, small-scale, single-center, and single-arm investigation, and no comparison with other methods was performed. Nevertheless, it is a technique that is not widely performed in interventional radiology suites, but it has high potential. On the basis of the results, a broader implementation of these techniques may lead to more comprehensive scientific evidence about their efficacy, and it could become a valuable weapon in the interventional radiologists’ arsenal, capable of providing, in a multidisciplinary setting, an additional therapeutic option for both complicated and natural biliary anatomy.

## 5. Conclusions

In conclusion, the treatment of percutaneous transhepatic lithotripsy using SpyGlassDSTM cholangioscopy has been demonstrated as efficient and safe for the treatment of difficult biliary stones. In some circumstances, PTL may be a good choice, especially for patients with changed anatomy or those who are not suitable candidates for more conventional endoscopic procedures like ERCP. Expanding research on this technique, particularly in prospective multi-institutional trials, could provide more definitive insights into its long-term efficacy and cost-effectiveness. Given the increasing application of SpyGlass cholangioscopy for various hepatobiliary and pancreatic disorders, its integration into standard clinical practice may continue to grow, offering minimally invasive alternatives to surgical interventions for complex cases.

This study has several limitations, including a small sample size, and the retrospective design of the study can introduce selection bias. Furthermore, there was no long-term follow-up data that can preclude the evaluation of long-term clinical outcomes. Future prospective research with larger cohorts and longer follow-up will be necessary for validating and expanding on these findings.

## Figures and Tables

**Table 1:** Summary of demographic characteristics and procedure results.

Patient Characteristic	Number of Patients	Percentage
Male sex	6	60%
Female sex	4	40%
Mean age (SD)	64 (22)	/
**Procedure Details**	**Number of Patients**	**Percentage**
Cholangitis due to gallstones	10	100%
History of hepatobiliary disease	2	20%
Chronic liver disease progressing to cirrhosis	1	10%
Secondary biliary cirrhosis post-liver transplant	1	10%
Intrahepatic lithiasis	3	30%
Extrahepatic lithiasis	7	70%
ERCP unsuccessful/contraindicated	6	60%
Intrahepatic stones with normal anatomy (no prior surgery)	4	40%

## Data Availability

The raw data supporting the conclusions of this article will be made available by the authors on request.

## Abbreviations

CBD	Common bile duct
CBDS	Common bile duct stone
D-SOC	Digital version of the Single Operator Cholangioscopy
EHL	Electrohydraulic system
ERCP	Endoscopic retrograde cholangiopancreatography
ESWL	Extracorporeal shock wave lithotripsy
MRCP	Magnetic Resonance Cholangiopancreatography
EUS-B	EUS-guided transhepatic biliary drainage
PTBD	Percutaneous transhepatic biliary drainage
PTL	Percutaneous transhepatic lithotripsy
